# Initial levels of mr-proadrenomedullin: a predictor of severity in patients with influenza a virus pneumonia

**DOI:** 10.1186/2197-425X-3-S1-A832

**Published:** 2015-10-01

**Authors:** F Valenzuela Sanchez, B Valenzuela Mendez, JF Rodríguez Gutierrez, R Bohollo de Austria, J Rubio Quiñones, L Puget Martínez, I Valiente Alemán, A Estella García

**Affiliations:** Hospital del SAS de Jerez, Critical Care Medicine, Jerez de la Frontera, Spain; Ginecology and Obstetric Department, Hospital Universitari Germans Trias i Pujol, Barcelona, Spain; Hospital del SAS de Jerez, Hematology, Jerez de la Frontera, Spain; Hospital Puerta del Mar, Critical Care Medicine, Cadiz, Spain; Hospital Santa María del Puerto, Critical Care Medicine, El Puerto de Santa María, Spain; Hospital Universitario de Puerto Real, Critical Care Medicine, Puerto Real, Spain

## Introduction

High levels of MR-proadrenomedullin (MR-proADM) have been described in critical sepsis patients. This is directly related to the relaxation of vascular tone and, therefore, hypotension and the presence of organ failure in patients with septic shock. In patients with severe pneumonia due to influenza A, although without great hemodynamic compromise, the presence of respiratory failure worsens the prognosis and significantly increases mortality.

## Objective

Evaluate the usefulness of MR-proADM comparing them to C-reactive protein (CRP) and procalcitonin (PCT) in the prognosis of patients with influenza A virus pneumonia.

## Methods

Prospective observational multicenter study. We included patients admitted to the ICU of five hospitals in Spain with the diagnosis of severe sepsis during a period of 36 months due to influenza A virus pneumonia. Biomarker levels (MR-proADM, CRP, PCT) were determined at admission. Data were compared with a control group (CG) of patients, also with influenza virus A pneumonia, but less severe who were not admitted to the ICU.

## Results

**Figure 1:**
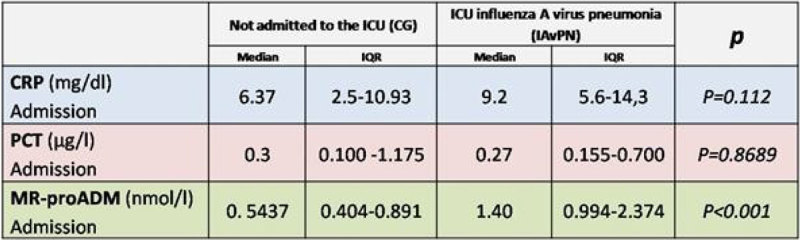
**Initial CRP, PCT and MR-proADM levels.**

66 patients were included: 41 patients with severe pneumonia caused by influenza A virus (IAvPN) and 25 patients were included in the control group (CG).The IAvPN group mortality was 29.26% (12/41). PCT levels were similar in both groups: 0.3 µg/l (IQR 0.00-1.175) in the GC and 0,27µg/l (IQR 0.155-0.700) in the IAvPN group. The levels of CRP at admission were 9.2 mg / dl (IQR 5.6-14,3) in IAvPN and 6.37 mg / dl (IQR 2.5-10.93) in the CG (p = 0.112). The MR-proADM levels at admission were 1.40 nmol / l (IQR 0994-2374) IAvPN against 0.5437 nmol / l (IQR from 0.404 to 0.891) in the CG (p = 0.001) (Figure 1).

**Figure 2:**
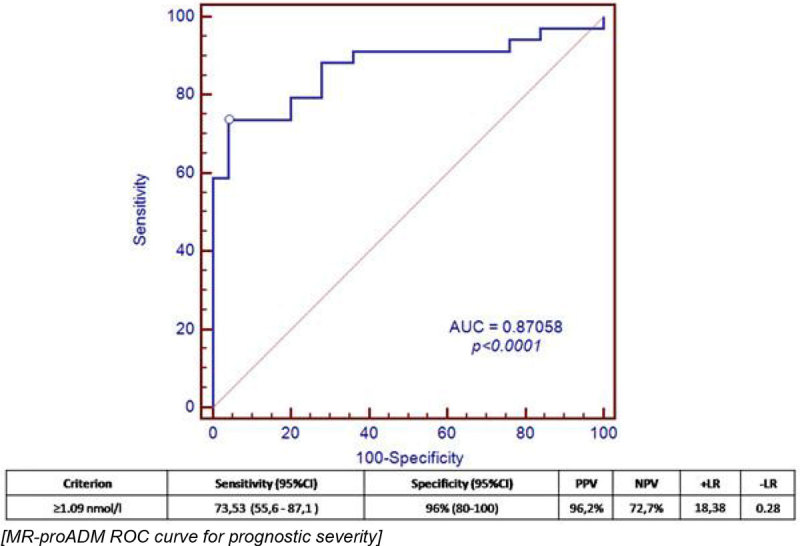
**MR-proADM ROC curve for prognostic severity.**

The area under the ROC curve (AUC) for prognostic severity (ICU admission) was 0.6769 (p < 0.0961) for CRP levels, 0.5767 (p < 0.543) for PCT levels and 0.87058 for MR-proADM levels (p < 0.0001). The optimal cutoff for severity (ICU admission) MR_proADM levels at admission was 1.09 nmol / l, with a sensitivity of 73.53% and a specificity of 96%.(Figure 2).

**Figure 3:**
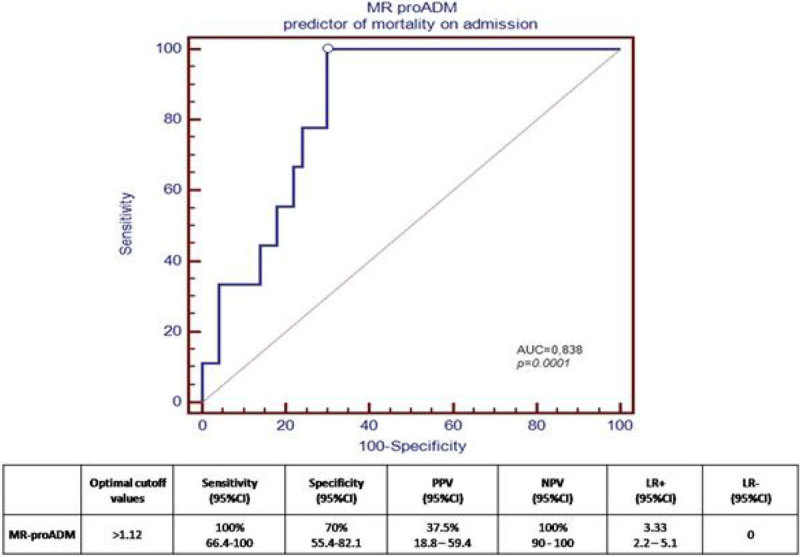
**proADM ROC curve for prognostic mortality.**

The non survivors showed greater MR-proADM levels with a median of 1.622 nmol / l (IQR 1.35-4.420) *vs* 0.8606 nmol/l (IQR 0.459-1.382) in the survivors (p = 0.0014). the PCT and CRP levels showed no significant difference in mortality groups. The AUC the ROC curve for prognostic mortality was: MR-proADM 0.838 (p = 0.0001); PCT 0.599 (p = 0.591) CRP 0.6400 (p = 0.0072) (Figure 3).

In the multivariate analysis (Cox proportional hazards models) only MR-proADM levels at admission, were statistically significant predictive factors for mortality in the ICU and at 90 days.(Table 1)

**Table 1:** multivariate analysis (Cox models)

Endpoint: 90-day mortality	Covariate	Hazard Ratio (95%CI)	Regression Coefficient	Standard error	p value
**Multivariate analysis (Backward Method)**	**MR-proADM at admission**	2.4931 (1.269-4.895)	0.9135	0.3460	***p = 0.0083***

## Conclusions

Initial MR-proADM levels are effective to determine the unfavorable outcome and the risk of ICU admission and mortality in patients with pneumonia due to influenza A virus.

